# Successful Management of a Parapharyngeal Abscess With Pharyngeal Perforation and Internal Jugular Vein Thrombosis in a Patient With a History of Lymphoma

**DOI:** 10.7759/cureus.80031

**Published:** 2025-03-04

**Authors:** Fernando Dip, Rene Aleman, Federico Marinelli, Alberto Rancati, Diego Sinagra

**Affiliations:** 1 Department of General Surgery, University of Buenos Aires, Buenos Aires, ARG; 2 Heart, Vascular &amp; Thoracic Institute, Cleveland Clinic Florida, Weston, USA

**Keywords:** deep neck space infections, non-hodgkin’s lymphoma, parapharyngeal abscess, pharyngeal perforation, thrombosis of the internal jugular vein

## Abstract

Deep neck space infections (DNSIs) are rare but potentially life-threatening conditions due to their rapid progression and proximity to vital structures. The conventional management of this condition is multidisciplinary, requiring broad-spectrum antibiotic therapy and surgical intervention when deemed necessary. The prognosis of DNSIs relies on the immune status of its host, severity, and anatomical location, with high and fluctuating mortality rates if inadequately managed. A timely intervention prevents the progression to higher-risk complications, including Lemierre syndrome, mediastinitis, and respiratory failure. The authors herein present the case of a complicated parapharyngeal abscess requiring a multidisciplinary medical and surgical approach via abscess drainage, pharyngeal defect repair, and muscle flap reinforcement. This case highlights the importance of early recognition, surgical expertise, and tailored postoperative care in the management of complex DNSIs.

## Introduction

Deep neck space infections (DNSIs) are uncommon yet hold significant risks due to the anatomical intricacies of the cervical region and the potential for severe complications, including Lemierre syndrome, mediastinitis, vascular thrombosis, and respiratory failure. Parapharyngeal abscesses are particularly challenging due to their proximity to vital structures such as the internal carotid artery (ICA), internal jugular vein (IJV), and cranial nerves. Any inadvertent surgical occurrence can lead to pharyngeal perforation and IJV thrombosis, consequently increasing the complexity of management [1,2]. Pharyngeal perforation is a rare complication of DNSIs and can result from increased intraluminal pressure secondary to a Valsalva maneuver. If present, a superimposed risk of infection spreading to adjacent spaces, systemic sepsis, and potential fistula formation can occur [3]. Moreover, when IJV thrombosis ensues, it can progress to advanced infections and is frequently associated with septic embolism or Lemierre syndrome [4,5]. This report describes the case of a patient with a history of lymphoma who developed a parapharyngeal abscess following the progression of an untreated sinus infection. The infection led to a pharyngeal perforation and IJV thrombosis, requiring urgent surgical intervention and multidisciplinary care.

## Case presentation

**Figure 1:**
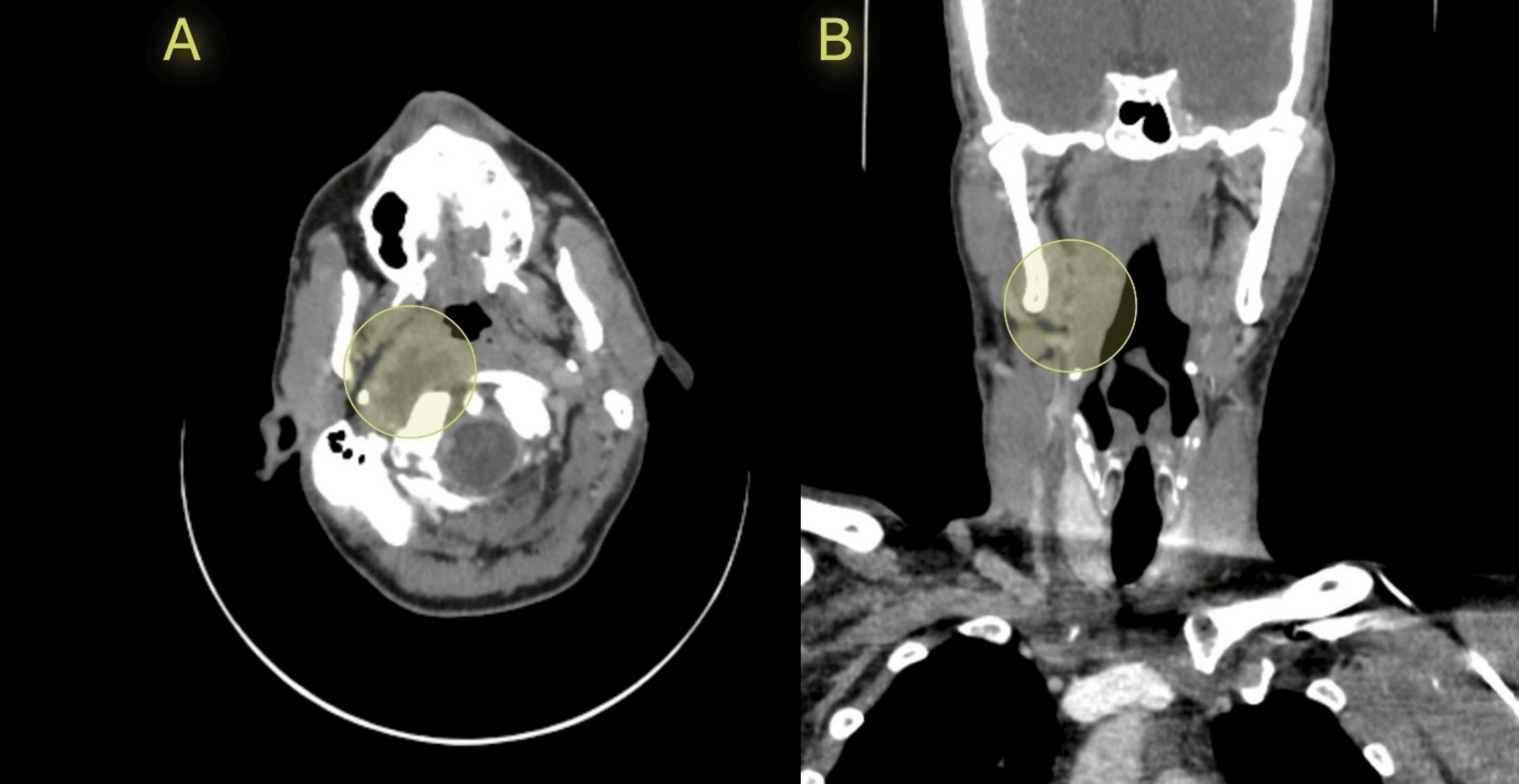
CT scan showing a large right parapharyngeal abscess (A) Axial view. Significant right parapharyngeal abscess located adjacent to the anterior border of the sternocleidomastoid muscle. (B) Coronal view.

A 47-year-old male presented to the emergency department with a history of progressive sinus congestion and nasal discharge, which had worsened over the past five days. The patient consulted medical attention following the onset of fever, general confusion, and severe right-sided neck pain. The patient reported no prior medical therapy for his sinus symptoms. His medical history was significant for non-Hodgkin’s lymphoma in remission for two years. The patient denied recent trauma; however, his symptoms were exacerbated after a forceful Valsalva maneuver while attempting to clear nasal congestion, leading to the sudden onset of severe throat pain and dysphagia. On admission, the patient was febrile, tachycardic, and in moderate respiratory distress. Physical examination revealed swelling and tenderness over the right cervical region with limited neck mobility. Oropharyngeal examination showed erythema and bulging of the posterior pharyngeal wall. Upon workup, the patient had a significant leukocytosis, elevated C-reactive protein, and hypoalbuminemia (Table 1). Blood cultures were drawn, and broad-spectrum antibiotics with piperacillin-tazobactam were initiated. Contrast-enhanced computed tomography (CT) of the neck showed a large fluid collection in the parapharyngeal space with free air, consistent with an abscess (Figure 1). Imagery was associated with a pharyngeal wall defect and the absence of contrast filling in the right IJV, confirming IJV thrombosis. Magnetic resonance imaging (MRI) excluded carotid artery involvement and mediastinal extension. Based on these findings, the patient was taken to the operating room for an abscess drainage and surgical repair of the pharyngeal perforation. Prior to intervention, verbal and written consent was given by the patient.

**Table 1:** Preoperative clinical evaluation and workup F, Fahrenheit; bpm, beats per minute; RR, respiratory rate; WBC, white blood cells

Preoperative clinical workup	
Temperature (°F)	102.9
HR (bpm)	112
RR	25
Systolic blood pressure (mmHg)	138
Diastolic blood pressure (mmHg)	81
Mean arterial pressure (mmHg)	100
WBC (µL)	17,200
C-reactive protein (mg/dL)	14.8
Albumin (g/dL)	2.9

Surgical management

Upon induction of general anesthesia, orotracheal intubation was performed under direct visualization, during which purulent material was observed in the oropharynx. A defect measuring approximately 2 cm was identified in the posterior pharyngeal wall. A cervicotomy was performed via an anterolateral approach with dissection anterior and medial to the sternocleidomastoid (SCM) muscle extending to the skull base through the parapharyngeal compartment. Approximately 60 mL of purulent material was drained, and cultures were obtained.

**Figure 2:**
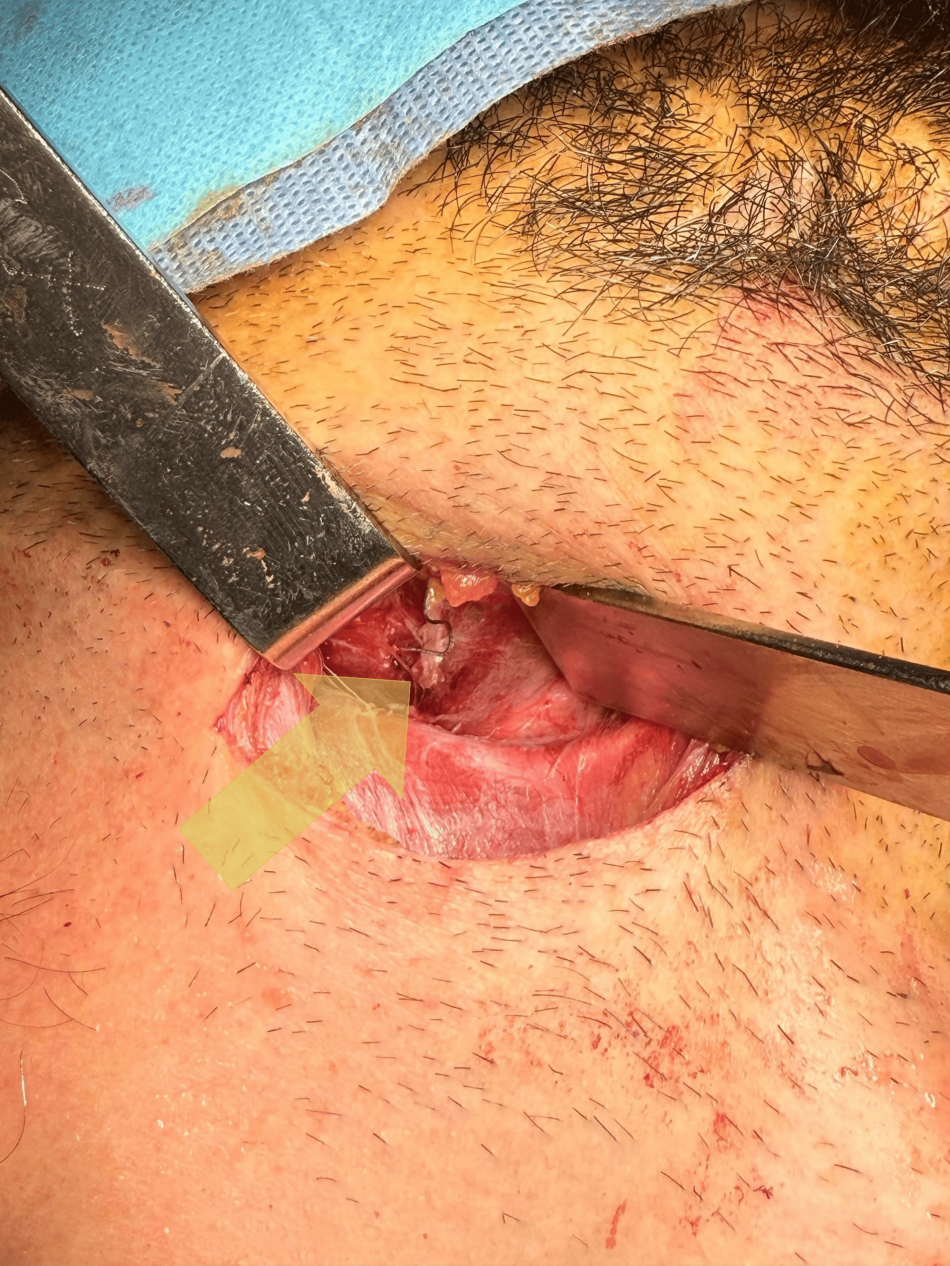
Surgical repair of the parapharyngeal perforation with sternocleidomastoid muscle.

The pharyngeal defect was repaired using a layered closure technique. The mucosal layer was closed with interrupted 4-0 polydioxanone (PDS) sutures, ensuring tension-free apposition of the edges. The muscular layer was reinforced with a pedicled SCM muscle flap, which was rotated into the defect to provide additional support and prevent fistula formation [1] (Figure 2). Hemostasis was achieved, a closed suction drain was placed in the parapharyngeal space, and a feeding tube was inserted transnasally for enteral nutrition during the postoperative period. The procedure was tolerated well and completed without complications. The patient was transferred intubated to the intensive care unit (ICU) for postoperative monitoring and management of systemic sepsis.

Postoperative course

The patient remained intubated for 24 hours to ensure airway protection and achieve hemodynamic optimization. Antibiotic therapy was continued and re-adjusted to ceftriaxone and metronidazole based on culture results, which isolated Streptococcus anginosus. On postoperative day 7, a methylene blue swallow test and contrast imaging with iodine-based dye were performed to assess the integrity of the pharyngeal repair. Both studies confirmed a fistula-free pharynx, leading to the patient's gradual transition to oral intake by postoperative day 15. The IJV thrombosis was managed with low-molecular-weight heparin as anticoagulation therapy, initiated after stabilization of the infectious process. Serial Doppler ultrasound scans were performed intermittently to confirm recanalization of the vein and isolate any evidence of thromboembolic complications [5].

By postoperative day 16, the patient was afebrile, tolerating a general diet, and showed no clinical signs of complications. He was discharged in stable condition with instructions for outpatient follow-up, including continued anticoagulation therapy and monitoring of the pharyngeal repair and thrombosis resolution.

## Discussion

The principles of approaching DNSI require a comprehensive knowledge of medical and surgical management to direct therapy in a timely fashion and halt a worsening progression. The present case illustrates a synergy of multidisciplinary therapies to address a challenging scenario where two high-risk complications ensue in DNSI. To ensure a successful outcome, the stepwise intervention required substantial medical coverage accounting for pathogen-directed antibiotic therapy, hemodynamic support and optimization, airway preservation, and restoration of physical and mechanical components of the affected area. Moreover, the medical and surgical approach displayed for this case emphasizes on the importance of timely intervention to improve the overall prognosis of the patient. Notably, the patient stabilization should be prioritized prior to any surgical conduct.

The occurrence of DNSIs is relatively uncommon, posing an annual incidence of 0.22 cases per 10,000 individuals, and an accompanying alarming increasing incidence rate over the last decade [6]. The rarity of its occurrence renders heterogeneous indications for directed therapy. Contemporary practice indicates that surgical drainage of an abscess secondary to DNSI is required in 10% to 83% of patients [1]. Failure to promptly address a DNSI can result in multiple life-threatening complications. Of these, IJV thrombosis is the most common complication, followed by mediastinitis and pharyngeal perforation. Owing to the scarce literature on pharyngeal perforation, this complication warrants careful attention. As it often derives from procedural techniques, all surgical interventions pertaining to DNSI should prompt extensive preoperative assessment to surgically navigate an anatomically challenging location.

The location of a DNSI serves as the surgical breaking point to avoid complications. When an abscess is identified in the lateral pharyngeal space, an external approach should be performed over an intraoral approach. The rationale holds sound in consideration of the neighboring anatomical landmarks that may be compromised. The external approach accounts for the presence of the carotid sheath within the lateral pharyngeal space and avoids surgical iatrogenesis. Conversely, in cases with compromised carotid artery, IJV, or mediastinum, a suitable approach is either a submandibular space approach or through the anterior aspect of the SCM muscle, which should be used when access is required to the IJV [7]. As a consequence of the IJV thrombosis present in this case, the authors opted for an SCM approach, which facilitated abscess drainage. Furthermore, this approach provided a surgical window to expeditiously create a muscular flap to repair the encountered perforation defect without further compromising the IJV.

By means of the muscular flap, no additional surgical intervention was required for the IJV thrombosis. All efforts were directed towards aggressive medical therapy including pathogen-directed antibiotics and adequate anticoagulation. The isolated etiology of the pathogen resulted in the modification of the antibiotic therapy and rapid control of associated symptoms. Furthermore, anticoagulation therapy provided an additional protective layer in the prevention of thromboembolic events and rapidly evidenced recanalization of the compromised vessels. Altogether, pharyngeal perforation has considerable rates of associated morbidity and mortality. Proper treatment focuses strategies towards thorough drainage of any source of infection, diversion of saliva and gastric fluids, and resumption of esophageal continuity as part of an early recovery at surgery protocol [8-10]. These were all measures directed in the management of this patient that led to a relatively rapid course of recovery.

This report demonstrates the successful management of a parapharyngeal abscess with pharyngeal perforation and IJV thrombosis in a patient with a history of lymphoma. Early diagnosis, timely surgical intervention, and a multidisciplinary approach were critical to achieving a favorable outcome. The authors instill the principle of early intervention and careful clinical evaluation in high-risk patients who are at risk of developing rare conditions, such as this case. While the measures were successful and mitigated the progression of the disease, the authors recognize the limitations of this case report. Firstly, the evidence base level is inherently low as it is a single case report [11-13]. Secondly, the incidence of both IJV thrombosis and pharyngeal perforation in DNSI are extremely rare and add to the complexity of its management. Lastly, the scarcity of clinical evidence and clinical guidelines convey a challenge for the operating surgeon as all interventions are made on a case-by-case basis. Ultimately, this case reports underscores the significance of addressing inadvertent DNSI and its complications. Addressing this premise can lead to substantial evidence and the creation of evidence-based clinical guidelines for the management of these rare complications.

## Conclusions

This case underscores the importance of a multidisciplinary approach to DNSIs, involving surgical expertise, targeted antimicrobial therapy, and intensive care support. While the patient’s medical history may have contributed to a heightened susceptibility to infection, the case was managed promptly and efficaciously. The display of the approach described herein emphasizes the need for vigilance in immunocompromised individuals, as they are prone to develop these complications. The management of rare occurrences, such as the ones present in this case, requires close evaluation and warrants further large cohort studies to draw definitive measures and standardize clinical guidelines.
